# A Real-Time Registration Algorithm of UAV Aerial Images Based on Feature Matching

**DOI:** 10.3390/jimaging9030067

**Published:** 2023-03-11

**Authors:** Zhiwen Liu, Gen Xu, Jiangjian Xiao, Jingxiang Yang, Ziyang Wang, Siyuan Cheng

**Affiliations:** 1 Faculty of Electrical Engineering and Computer Science, Ningbo University, Ningbo 315211, China; 2 Ningbo Institute of Materials Technology and Engineering, Chinese Academy of Sciences, Ningbo 315201, China

**Keywords:** SuperGlue, feature matching, drone, real-time image registration, image blocking, target geolocation

## Abstract

This study aimed to achieve the accurate and real-time geographic positioning of UAV aerial image targets. We verified a method of registering UAV camera images on a map (with the geographic location) through feature matching. The UAV is usually in rapid motion and involves changes in the camera head, and the map is high-resolution and has sparse features. These reasons make it difficult for the current feature-matching algorithm to accurately register the two (camera image and map) in real time, meaning that there will be a large number of mismatches. To solve this problem, we used the SuperGlue algorithm, which has a better performance, to match the features. The layer and block strategy, combined with the prior data of the UAV, was introduced to improve the accuracy and speed of feature matching, and the matching information obtained between frames was introduced to solve the problem of uneven registration. Here, we propose the concept of updating map features with UAV image features to enhance the robustness and applicability of UAV aerial image and map registration. After numerous experiments, it was proved that the proposed method is feasible and can adapt to the changes in the camera head, environment, etc. The UAV aerial image is stably and accurately registered on the map, and the frame rate reaches 12 frames per second, which provides a basis for the geo-positioning of UAV aerial image targets.

## 1. Introduction

In light of the rapid development of UAV technology [1,2,3], the onboard camera of the UAV is often used to identify and locate the target. The maturity of feature-matching [4,5,6,7] technology means that it is often used for target tracking and positioning. By registering the UAV camera image containing the target on the geographic map or the satellite plane projection map, the real-time geolocation of the camera screen target can be determined, and the effect of augmented reality can be achieved. Therefore, it is of great significance to study the real-time registration technology of the UAV aerial images and maps.

However, for low-texture and high-resolution maps, the rapid movement of the UAV and transformation of the camera’s pan and tilt behavior render feature matching between the map and UAV aerial image difficult. To solve the abovementioned problems, in this paper, we propose a registration algorithm based on SuperGlue [8] and hierarchical block. The algorithm can adapt to the changes in scene and camera pan and tilt behavior, reduce the difference between the map and UAV aerial image, and accurately register the UAV aerial image on the map with sparse texture in real time.

## 2. Related Work

Concerning traditional feature detection algorithms, in 1999, Lowe, D G. proposed the Sift [9] algorithm of local scale invariant features, which is one of the classic, most traditional features capable of stable detection in regard to image rotation, blur, different scales, and brightness. In 2006, the Surf [10] algorithm, proposed by H. Bay, was found to be equivalent to the accelerated version of the Sift algorithm. With the aim of maintaining the original performance of the Sift algorithm, it solved the shortcomings of the high computational complexity and long-term consumption of the Sift algorithm. However, using the Sift and Surf algorithms, it is still impossible to conduct real-time feature-matching tasks for UAV camera images. In 2011, Rublee et al. proposed the Orb [11] algorithm as an effective alternative to Sift and Surf. Orb used the Fast [12] algorithm as the basis for feature extraction and the BRIEF [13] algorithm as the basis for feature matching. The computation time of the Orb algorithm was 1% of that of Sift and 10% of that of Surf, but the feature extraction and matching effect, in the case of low-texture scenes, were poor, and the accuracy was low.

In work aiming to improve the accuracy and speed of feature extraction and matching algorithms, due to the tilt and large angle of view, some low-altitude UAV aerial images are difficult to register accurately. To solve this problem, Wang et al. [14] used the improved ASIFT(affine scale-invariant feature transform) algorithm to collect the initial feature points, the Weighted Least Squares Matching (WLSM) method to correct the positioning of the feature points, and the adaptive normalized cross-correlation algorithm to estimate the local transformation model. Finally, the UAV aerial images with large changes in perspective could be registered at the sub-pixel level. Liu et al., stitching high-resolution UAV aerial farmland images [15], found that the image was down-sampled before the detection of the features, aiming to reduce the number of feature points, and the feature matching was realized by a feature descriptor based on gradient normalization. The Progressive Sample Consistency algorithm was used to eliminate the false matching points, which improved the speed and accuracy of the algorithm. Wu et al. [16], stitching forest images taken by a UAV, found that high scene similarity leads to low accuracy in feature matching and a long stitching time. To solve this problem, the arccosine function ratio of the unit vector dot product was introduced so as to overcome the long matching time caused by the excessive number of matching points, and the Fast Sample Consistency (FSC) algorithm was introduced to eliminate the false matching points, which improved the accuracy of the algorithm. However, the abovementioned methods may not obtain satisfactory feature-matching results for high-resolution, low-texture maps and UAV aerial images and are far from capable of performing real-time tasks. Goh, J. N. et al. [17] introduced matrix multiplication into the Ransac [18] algorithm for the task of the real-time stitching and mapping of UAV aerial images, which greatly reduced the processing time for calculating the homography matrix. Moreover, in the stitching process, several input images were divided into two halves to reduce the time for feature detection and extraction. Xiong, P. et al. [19], conducting real-time UAV stitching, used the prediction region to match the features of the current image, ensuring that the time required for the task was the same and reducing the stitching error. Zhang, G. et al. [20] introduced the semantic segmentation [21,22,23] algorithm to filter the foreground features, which improved the robustness and limitations of the algorithm, in order to solve the problems of misalignment and tearing caused by the significant changes in the dynamic foreground during the real-time splicing of the UAV images. However, the segmentation algorithm may lead to the degradation of the real-time performance.

In terms of the registration of UAV images and maps, Yuan, Y. et al. [24], aiming to solve the problem that the UAV aerial images and Google Maps cannot be accurately registered due to large differences in the viewpoint direction, shooting time, and height between images, obtained Google Map images from the approximate position of the UAV aerial images. Using the VGG16 [25] model to extract deep convolutional features, the algorithm achieved a more robust and effective registration effect. Zhuo, X. et al. [26] stated that the greatest challenge in matching UAV aerial images with previously captured geo-referenced images is the significant differences in scale and rotation. They proposed dense feature detection and one-to-many matching strategies, combined with global geometric constraints for outlier detection, to identify thousands of valid matches in cases where Sift failed. This method can be used to realize the geo-registration of UAV aerial images, and the registration accuracy reaches the decimeter level. However, the algorithm was only studied in terms of its accuracy and was not optimized in real time. In order to avoid the error accumulation and distortion caused by using local methods to stitch continuous images captured by UAV airborne cameras, Lin Y. et al. [27] proposed using a high-resolution map as a reference image, to register frames on the map and perform stitching by the frame-to-frame registration method. Nassar A. et al. [28] realized the positioning of the UAV by registering the forward- and downward-view images taken by the UAV and the satellite map. The algorithm only used the airborne camera and did not require GPS. The semantic shape-matching algorithm was introduced in the registration process to improve the accuracy, which proved that the utilization of visual information can provide a promising method of UAV navigation.

Nowadays, feature-matching algorithms have powerful functions and are often used for image stitching [29,30,31], positioning, mapping, registration, and other visual tasks. However, using this technology for scenes with sparse textures and tasks requiring a high real-time performance and accuracy remains challenging.

The main work reported in this paper is as follows:

1. The SuperGlue matching algorithm was applied for the real-time registration of UAV aerial images and maps, and a hierarchical blocking strategy combined with prior UAV data is proposed here to optimize the performance of the algorithm.

2. The inter-frame information was integrated into the matching process to improve the stability of the algorithm.

3. A method for updating map features in real time is proposed to improve the robustness and applicability of the algorithm.

## 3. Materials and Methods

### 3.1. Overall Design Framework

**Figure 1:**
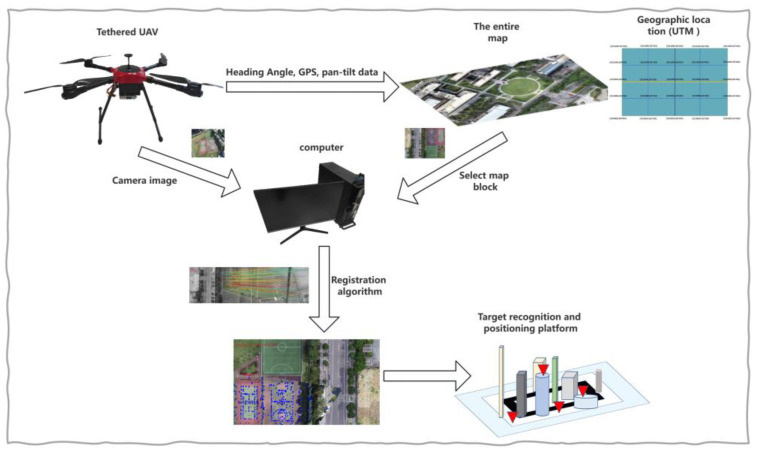
System functional architecture.

The functional architecture of the system is shown in Figure 1. It is mainly divided into four platforms, including the UAV airborne terminal, the map terminal with the geographical location, the processing platform responsible for registration, and the target recognition and positioning platform. The map terminal is divided into multiple layers and blocks and has accurate geographical coordinates. The geographical coordinates adopt the Earth plane coordinate system (UTM coordinates). The UAV terminal provides the altitude, heading angle, rotation angle of pan-tilt-zoom camera, GPS, and other data to the map terminal, and the map terminal selects the corresponding map block based on this information. After the accurate registration of the UAV aerial image and the map block, the UAV aerial image also contains information on the geographical position, and the corresponding transformation relationship is sent to the target positioning platform for the geo-positioning and remapping of the target and other applications. In this paper, we mainly study the registration algorithm for the map and the UAV image. In the study, the abovementioned processes were carried out in real time.

**Figure 2:**
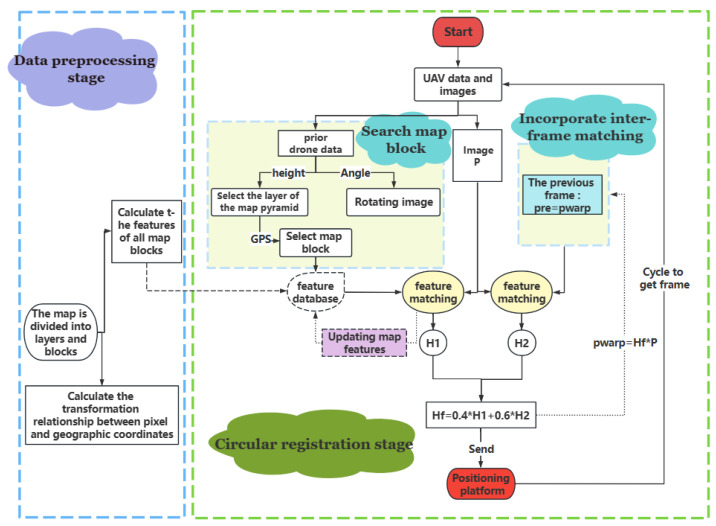
Overall design flow chart.

The overall design flow chart of the algorithm is shown in Figure 2, which mainly includes the stages of the pre-generation of the image features and other data, the automatic search of the map blocks combined with the prior UAV data, the integration of the inter-frame information module, and the real-time update module of the map features.

### 3.2. Hierarchical Blocking Strategy Combined with Prior UAV Data

**Figure 3:**
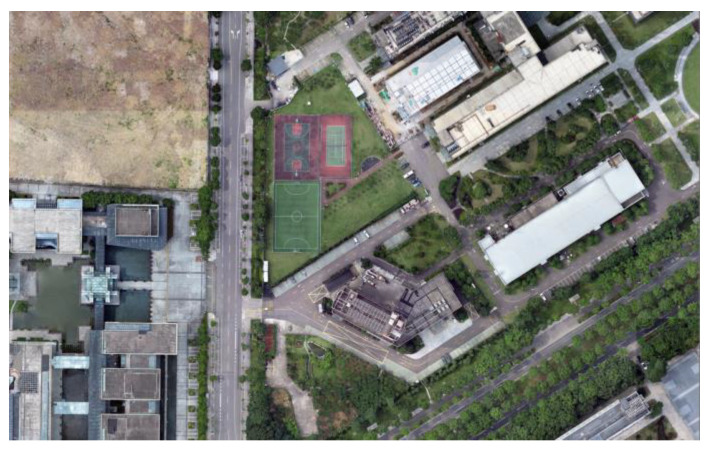
Orthographic projection map with geographic information.

Through the real-time feature matching of the video picture from the UAV’s airborne camera with the map, the camera picture can be accurately registered to the map (the map is an orthographic projection of the satellite perspective generated by CC software, as shown in Figure 3). The map is manually calibrated, and the transformation relationship between the pixel and geographic coordinates for the map is as follows:

(1) g=H×p

where g is the Utm coordinate, p is the pixel coordinate, and H represents the transformation matrix, which can be obtained by manually calibrating 4 pairs of points. Following the registration, the geographical position of the target can be obtained using the pixel coordinates of the target in the UAV aerial image, and the geolocation function can also be realized. However, it is difficult to accurately register the dynamically changing camera images through feature matching with a wide range of maps. For this problem, our solution is to divide the map into blocks to obtain a number of local maps, and to combine the prior UAV data to flexibly select the local maps to be matched.

#### 3.2.1. SuperPoint and SuperGlue Feature-Matching Algorithms

The first step of the feature-matching task is the extraction of the feature points. Feature points refer to the positions of 2D image points that can be stably and repeatedly detected under different lighting conditions and different viewpoints. SuperPoint [32] is a type of deep learning feature that designs a self-supervised network framework. Compared with the patch-based method, it can simultaneously extract the location of feature points and the descriptors on the original image with pixel-level accuracy. It is suitable for ensemble computer-vision-matching tasks, such as homography estimation.

**Figure 4:**
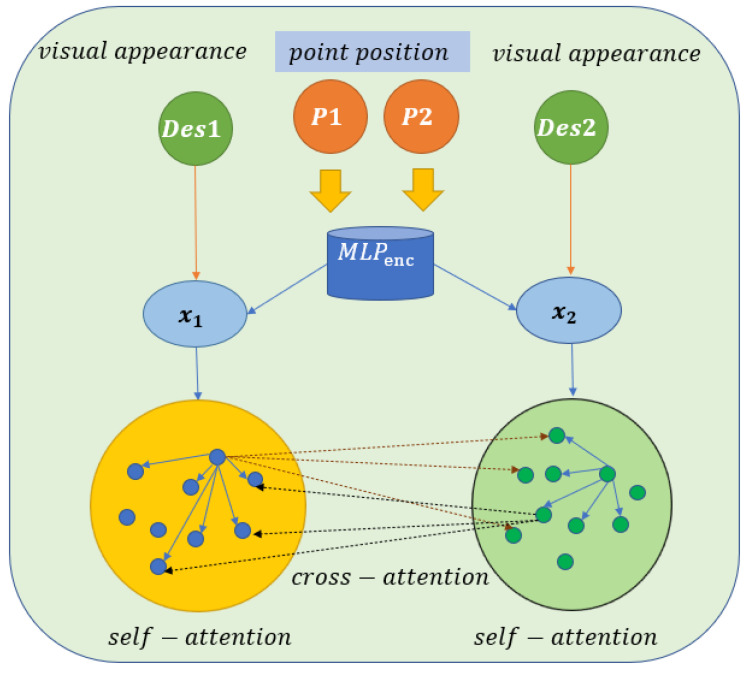
Attentional Graph neural network (adapted from ref. [8]).

SuperGlue is a real-time feature-matching algorithm based on a graph neural network [33], which can filter outliers while performing feature matching. Feature matching is conducted by solving the differentiable optimal transfer problem. Compared with the traditional, hand-designed features, it can achieve the best results in indoor and outdoor environments and achieve real-time feature matching on GPU. Its inputs are the feature points and descriptors of the two images to be matched, and the output is the matching relationship between the features of one image and the features of another image. In this process, two kinds of attention [34] mechanisms are introduced: 1. self-attention, which serves to enhance the acceptance of local descriptors and 2. cross-attention, where the image is matched by approximate back-and-forth observation. The Attentional Graph neural network, the first component of the SuperGlue network, is shown in Figure 4. The component is divided into two key technologies. The first serves to embed the key point position into the high-dimensional vector using multi-layer perception (MLP) [35] and then fuse the information on its visual appearance. The initial representation of each key point combines the visual appearance and position and is expressed as follows:

(2) $$x_{i}^{0}=d_{i}+MLP_{enc}\left(P_{i}\right)$$

where $x_{i}^{0}$ is the initial representation of key point i, $d_{i}$ is the visual appearance of key point i, $P_{i}$ is the location of the key point, and $MLP_{enc}$ means that multi-layer perception is used to increase the dimension of the feature.

The second form of technology used is the attention mechanism (cross/self + MLP), which serves to calculate an increment (delta0 or delta1) of the descriptors encoded by the key encoder (des0 and des1) in order to update the descriptors. If the mechanism is self-attention, the (attention + MLP) layer is passed into des0 and des0, and if the mechanism is cross-attention, the layer is passed into des0 and des1. The formula is as follows:

(3) $${\;}^{\left(l+1\right)}x^{AorB}={\;}^{\left(l\right)}x^{AorB}+MLP([{\;}^{\left(l\right)}x^{AorB}||m_{\epsilon }])$$

where ${\;}^{\left(l+1\right)}x^{AorB}$ represents the des0 or des1 to be updated, ${\;}^{\left(l\right)}x^{AorB}$ represents the current des0 or des1, [·||·] represents the concatenation operation, and $m_{\epsilon }$ represents the result of the aggregation of self- and cross-information. SuperGlue is one of the best feature-matching algorithms based on deep learning.

In this study, the SuperPoint and SuperGlue algorithms were used to perform feature matching. Although the traditional Sift and Surf have high accuracy, they are not real-time algorithms. Orb is a real-time and commonly used algorithm in research; however, the robustness of the Orb algorithm is poor in some scenes, it produces only a single color or sparse texture. Compared with the Orb algorithm, the SuperPoint and SuperGlue algorithms produce better robustness and accuracy results for sparse texture scenes, can extract more feature points, and have a higher matching accuracy.

#### 3.2.2. Hierarchical and Block Strategy

**Figure 5:**
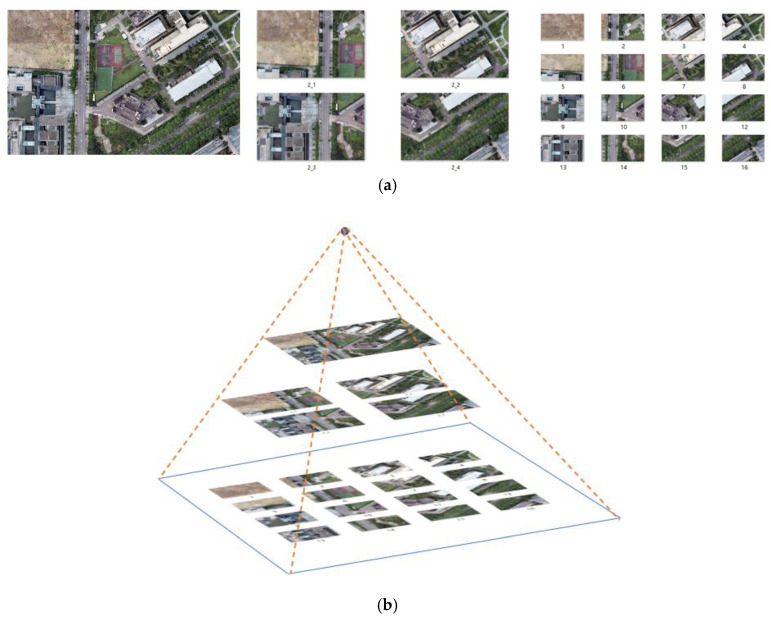
A map with layers and blocks, the numbers in Figure represent the numbers of the map blocks: (**a**) plan diagram; (**b**) map pyramid.

Due to the map’s high resolution and wide geographic coverage, it is not feasible to directly match the camera footage with the entire map. Moreover, most of the areas in the map are irrelevant to the UAV aerial images, which leads to an increased time cost and lower-accuracy results when feature matching is performed. If one selects an area in the map that is roughly the same as the camera image for matching, more matching points can be generated, and the accuracy and speed of the matching can be greatly improved. In this study, the map was divided into blocks, and a pyramid was constructed in layers so that the most appropriate block area could be selected each time for matching with the camera image, thus ensuring high accuracy, real-time matching, and reducing the large number of mismatches. The specific hierarchical blocks are shown in parts (a) and (b) of Figure 5.

Through clipping and downsampling techniques, we set the resolution of all map blocks to 1920 × 1080, and the number of map blocks that were positioned closer to the top level was low. The upper level of the pyramid is not divided, and it contains the largest field of view. It is suitable for matching when flying at high altitude or when the field angle of the airborne pan–tilt–zoom camera is large. After dividing the map into blocks, one can pre-calculate the SuperPoint features of all the blocks and store them in the feature array and pre-calculate the transformation relationship between the geographical coordinates and pixel coordinates of all the map blocks. A homography matrix array can be used for storage. For each camera image to be matched, only the feature of this frame must be calculated. The sequence number of the map block must be determined during matching, and the corresponding feature must be selected from the feature database for matching, which can accelerate the matching process.

#### 3.2.3. Automatic Map Block Search Strategy Combined with Prior UAV Data

**Figure 6:**
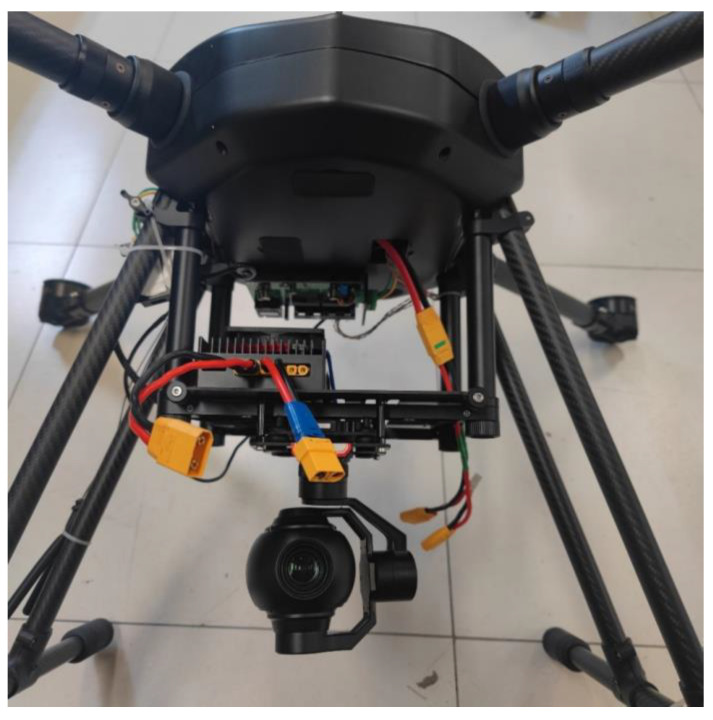
Pan–tilt–zoom camera (mounted under the UAV).

**Figure 7:**
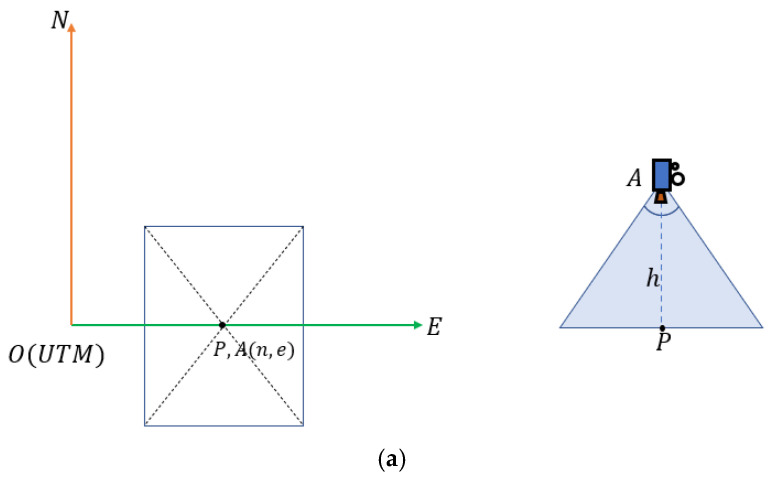

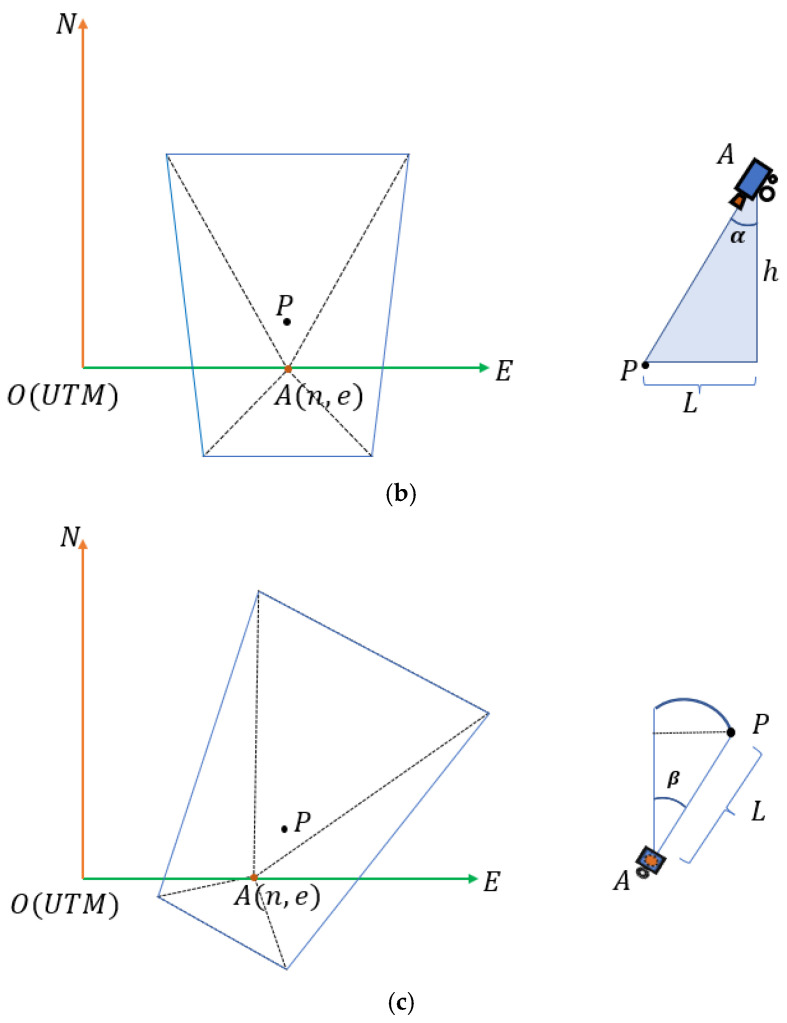
Top view of the camera’s field-of-view.  A(n,e) represents the camera position and coordinates, P represents the center point of the camera image, h is the altitude of the UAV, and L represents the displacement of P when the camera rotates up and down. (**a**) Camera without rotation; (**b**) camera is rotated up and down by α degree; (**c**) camera is rotated up and down by degrees and left and right by β degrees.

When the UAV operates at a high altitude, the height information of the UAV is used to select the layer of the map pyramid (20–40 m selected as the third layer, 40–80 m selected as the second layer, and higher than 80 m selected as the top layer). Through the UAV GPS and rotation angle information of the camera, the geographic coordinate (Utm coordinate) of the center of the current camera image can be roughly calculated. Figure 6 shows a pan–tilt–zoom camera and its mounting position; the camera can be rotated left and right or up and down. First, the UTM coordinate of the position directly below the UAV can be obtained via GPS (the UTM coordinate and GPS information can be converted to each other). Then, the UTM coordinates of the center of the camera image can be estimated from the camera rotation angle information. Figure 7 shows the geometric diagram of the camera field-of-view in three cases. The formula is as follows:

(4) L=h×tanα

where h represents the altitude of the UAV, α represents the pitch angle of the camera, and L represents the displacement of the image center after the camera is rotated up and down.

(5) $$P_{utm}=\left(n+L\times cos\beta ,e+L\times sin\beta \right)$$

Here, (n,e) represents the UTM coordinate of the position directly below the UAV, which can be converted directly by GPS, β represents the yaw angle of the camera, and $P_{utm}$ represents the approximate UTM coordinate of the center of the current camera image. After the $P_{utm}$ is obtained, the map block containing this coordinate is selected, and finally, the features of the map block in the feature array are selected for feature matching with the UAV aerial image.

#### 3.2.4. Rotation

Since the matching performance of the SuperGlue algorithm decreases when the angle between the map and the camera picture is 45 degrees or greater, when the angle between the two is above a certain threshold (in our study, based on the empirical values, we set the threshold to 25 degrees), the image of the camera must be rotated beforehand and then matched with the map. The specific angle of rotation is determined by the yaw angle of the unmanned aerial vehicle itself. After the rotation correction, the matching effect is greatly improved. The image rotates around the center point of the image, and the rotation matrix is R. The formula is as follows:

(6) $$A=\left[\begin{array}{ccc} 1 & 0 & -a\\ 0 & 1 & -b\\ 0 & 0 & 1 \end{array}\right]\;\cdot \;B=\left[\begin{array}{ccc} cos\theta & sin\theta & 0\\ -sin\theta & cos\theta & 0\\ 0 & 0 & 1 \end{array}\right]\;\cdot \;C=\left[\begin{array}{ccc} S & 0 & 0\\ 0 & S & 0\\ 0 & 0 & 1 \end{array}\right]\;\cdot \;D=\left[\begin{array}{ccc} 1 & 0 & a\\ 0 & 1 & b\\ 0 & 0 & 1 \end{array}\right]\;R=D\times C*B\times A$$

The transformation relationship between the pixel and geographical coordinates of the UAV aerial image is as follows:

(7) $$g=H_{i}\times H_{f}\times R\times P$$

where $H_{i}$ is the transformation relationship between the pixel and geographical coordinates of the selected map block (i represents the number of the map block),$\;H_{f}$ is the result of the feature matching, R is the rotation matrix, P is the pixel coordinate of the UAV aerial image, and g is the geographical coordinate.

### 3.3. Inter-Frame Information Fusion

#### 3.3.1. Inter-Frame and Global Matching Fusion

**Figure 8:**
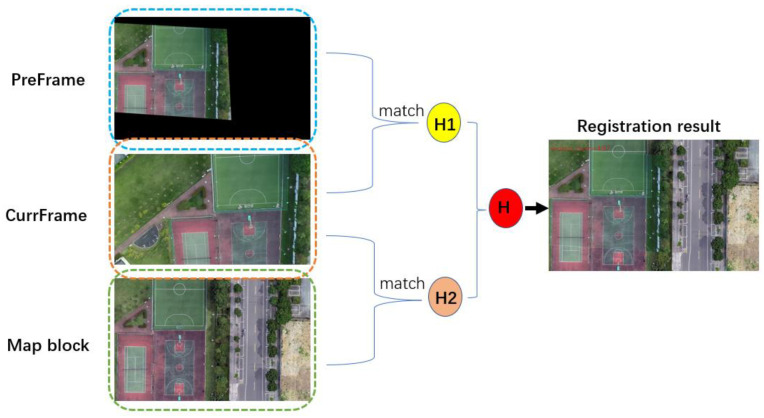
Weighted fusion of inter-frame and global matching in the figure, PreFrame represents the transformed previous frame and CurrFrame represents the current frame.

When each frame is matched with the map block, the global registration of the matching points is diminished, and the accuracy is low so that the registration of each frame is not coherent and the registered frame appears to be affected by high-frequency jitter. This process can be made more stable by integrating inter-frame matching, since both adjacent frames are slightly shifted. The current frame image is matched with the map and the transformed previous frame simultaneously, two homography transformation matrices are calculated, respectively, and then the two are weighted and fused to obtain the final homography transformation matrix, as presented in Figure 8. The integration of inter-frame matching creates a smoother and more stable registration process, without producing obvious jitter. The appropriate formula is as follows:

(8) $$H_{f}=w1\times H1+w2\times H2$$

where H1 is the homography matrix matched with the transformed previous frame, H2 is the homography matrix matched with the map, w1 and w2 represent the weights (in our study, we set w1 to 0.4 and w2 to 0.6), $H_{f}$ is the transformation matrix of the current frame.

#### 3.3.2. Anomaly Matrix Detection and Removal

When there are not enough matching points, the error of the calculated homography transformation matrix is too large, and the registration effect is very poor. The threshold can be set according to the empirical value in order to filter the small set of matching points. Because the motion of the screen is smooth, the homography transformation of the previous frame can be used to solve the problem of occasional registration anomalies. The rules are as follows:

(9) $$H_{f}=\{\begin{array}{c} H_{pre}\;\;,\;\;\;\;\;\;\;\;\;\;\;\;\;\;\;\;\;\;\;\;\;\;\;\;\;\;\;\;\;\;\;\;\;\;\;\;\;\;if\left(mkpt1.size\;\leq 25\right)\\ findH\left(mkpt1,mkpt2\right)\;\;,\;\;\;\;\;if(mkpt1.size>25) \end{array}$$

where $\;H_{pre}\;$ is the transformation matrix of the previous frame, findH represents a function that uses the Ransac [18] algorithm to obtain a transformation matrix, mkpts1 is the matching point of the map or previous frame, mkpts2 is the matching point of the current frame, and $H_{f}\;$ is the transformation matrix of the current frame.

### 3.4. Map Feature Update

Usually, when the UAV image is in a difficult matching area, the registration effect is poor. For example, when the center point of the airborne camera image is at the edge of the matching map block, the area of overlap between the map block and the camera image is relatively small, and large registration errors can easily be produced due to the lack of matching points during registration. When the camera image is in a low-texture scene or the camera image is tilted at a large angle, feature matching with the map block is more difficult, and it is difficult to achieve a good registration accuracy. Here, to address the abovementioned problems, a method of updating the map features is proposed, which uses the features of the UAV screen to update the map features in real time and solves the problem of the immutable map’s significant limitations, enabling it to adapt to the changing scene and automatically update its own features following the changes in the scene. Compared with the immutable map, it has a stronger robustness and adaptability. (The scene is not always the same, being affected by sunlight and weather. The map texture information is not especially rich in terms of color, texture, and brightness, and it is significantly different from the camera image.) The rules for the feature update are as follows:

(10) $$fea_{m}=\{\begin{array}{c} \{\begin{array}{c} des_{m}=des_{f}\\ score_{m}=score_{f}\\ keyPoint_{m}=keyPoint_{m} \end{array}\;\;,\;\;\;\;\;\;conf\geq 0.6\\ fea_{m}\;\;\;\;\;\;\;\;\;\;\;\;\;\;\;\;\;\;\;\;\;\;\;\;\;\;,\;\;\;\;\;\;conf<0.6 \end{array}$$

where $fea_{m}$ represents the SuperPoint feature in the map (SuperPoint features include key point position, feature descriptor, and feature probability), $des_{m}$ and $des_{f}$ represent the feature descriptor of the map and UAV aerial image, respectively, $score_{m}$ and $score_{f}\;$ represent the feature probability, $keyPoint_{m}$ represents the key point position of the map, and conf represents the confidence of a pair of matches.

When a frame of the UAV aerial image is matched with the map, the feature points of the frame image with a matching confidence value higher than 0.6 are selected to cover the features of the corresponding feature points in the map, including the feature descriptor and probability, and the position of the feature point remains unchanged.

## 4. Experimental Results

The experiment was mainly divided into four parts. One compared the performance of the proposed and Orb methods in two aspects: feature-matching and registration effect. The other verified the effectiveness of several improvements proposed in this paper; a vertical comparison experiment was conducted.

The vertical comparison experiment can be divided into three aspects. Firstly, the feature-matching effect prior to and following map blocking and rotation activity was compared. Secondly, the stability of the registration prior to and following integrating the matching information between frames was compared. Finally, the accuracy of registration prior to and following the real-time updating of map feature points was compared, and the evaluation was conducted considering subjective and objective perspectives.

**Figure 9:**
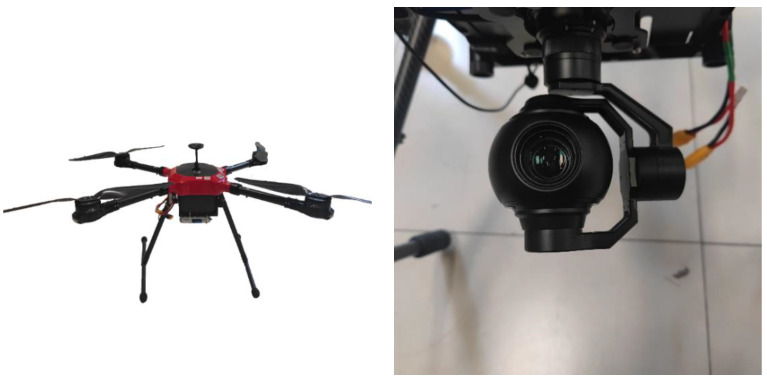
The multirotor X-type tethered UAV and pan–tilt–zoom camera.

The multirotor X-type tethered UAV (with a pan–tilt–zoom camera, as depicted in Figure 9) was used in the present experiment, the resolution of all map blocks was 1920 × 1080, the resolution of the UAV aerial image was 1920 × 1080, and the confidence threshold of the SuperGlue algorithm was set to 0.2.

### 4.1. The Effect of the Proposed Method and the Orb Algorithm

This experiment mainly compared the traditional Orb-matching algorithm with the method proposed in this paper, and the Orb algorithm used the BF [36] algorithm to conduct the matching. Two groups of map blocks and UAV aerial images were selected to compare the matching effect and accuracy of the matching-point pairs of the two methods (the Ransac [18] algorithm was used to calculate the matching accuracy in the experiment). Then, the registration results of the two methods were compared, where the registration result refers to overlaying the registered UAV aerial images onto the map block.

**Figure 10:**
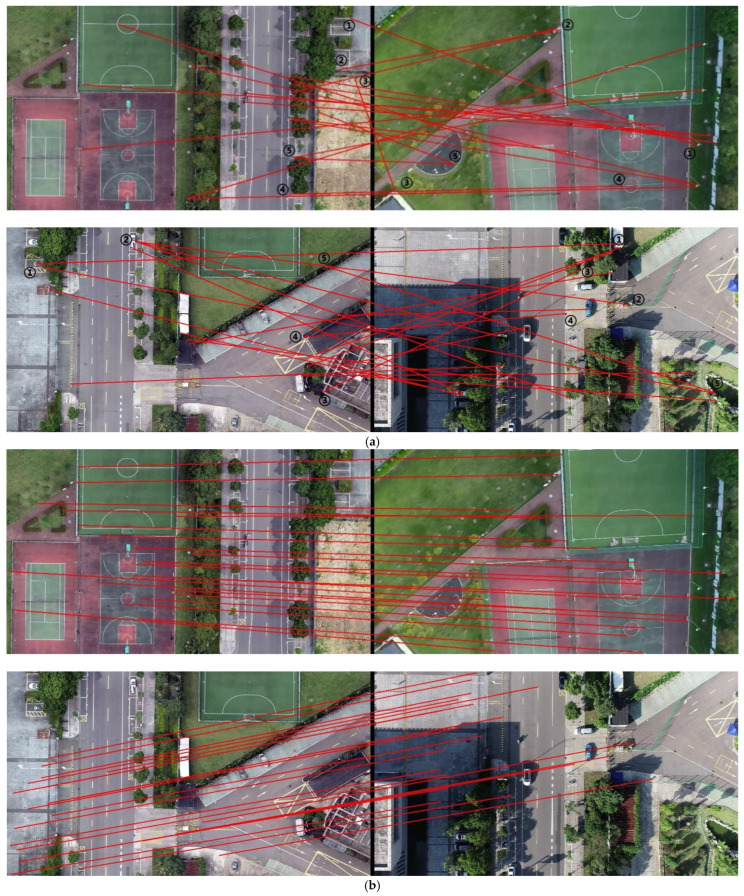
Comparison of feature matching between the Orb and SuperGlue algorithms: (**a**) Orb feature matching (left: map, right: camera, the numbers in the figure represent incorrect matches); (**b**) SuperGlue feature matching (left: map, right: camera).

In Figure 10, we presented the effect of feature matching between the Orb algorithm and our proposed method. In order to present clearer results, we uniformly selected 20 pairs of matches and drew them. From the figure, it can be observed that the Orb algorithm has many incorrect matches (we selected five of them to mark). Similarly, we also uniformly selected 20 matching points for the SuperGlue algorithm to be drawn, and we can observe that basically no error matching is evident.

Table 1 presents the comparison of the number and accuracy of matching-point pairs of the two methods. From the two groups of experiments, we can observe that the Orb algorithm and our method can attain a relatively high number of matching-point pairs; however, after eliminating the mismatching-point pairs by the Ransac [18] algorithm, the remaining correct matching-point pairs of the Orb algorithm are very few. The table also shows that the matching accuracy of the Orb algorithm is very low, indicating that most of the matching-point pairs obtained by the Orb algorithm are invalid.

**Figure 11:**
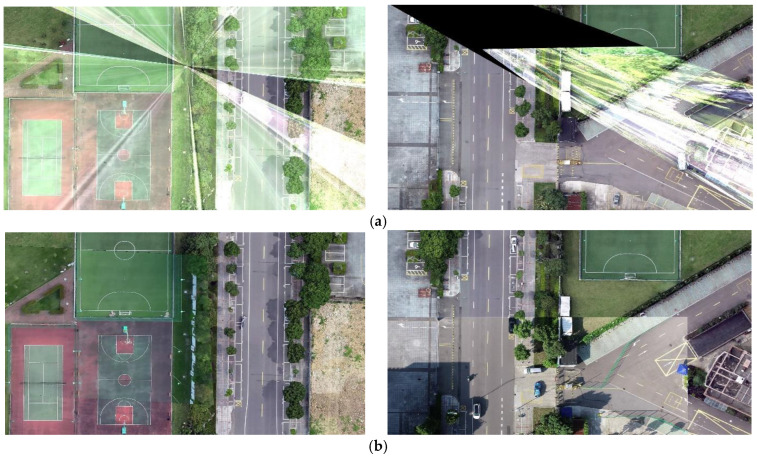
Registration effect: (**a**) Orb algorithm; (**b**) our proposed method.

In Figure 11, we present two groups of image registration results for the Orb algorithm and our method. It can be observed that our method can accurately register the UAV aerial images and maps; however, the Orb algorithm cannot register the two objects. It can also be observed from the figure that when the Orb algorithm was used, an abnormal result was obtained, which was caused by the incorrect matching of the Orb algorithm, because the homography transformation matrix calculated using the incorrect matching method was also wrong.

### 4.2. Blocking and Rotation Experiments

This experiment can be divided into two aspects. The first verified that the map has a better feature-matching effect with the UAV aerial image after dividing it into blocks. We selected a recorded aerial video of the UAV, a map block, and a non-block map (with a greater geographical range), and we matched the features of the video frame images with the two maps, respectively. The effect of the feature-matching process was evaluated by the number of matching points, and we also compared their running speed.

The second aspect involved verifying that the UAV aerial image had a stronger feature-matching effect when it was rotated to face the same direction as the map. Similarly, we selected 10 frames of the UAV aerial images that were not consistent with the map direction, and we rotated them by the heading angle of the UAV to obtain a set of images that were consistent with the map direction. Feature matching between these images and the map was performed, and the effect of feature matching prior to and following rotation was evaluated by the number of matching points obtained.

**Figure 12:**
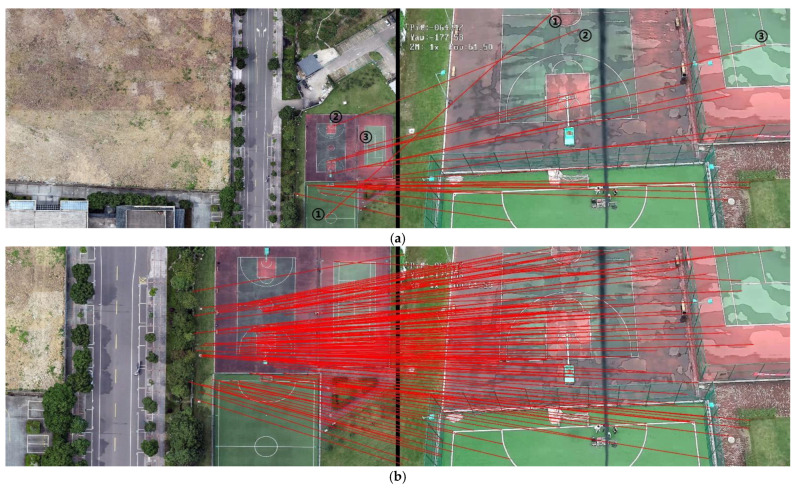
Comparison of the matching effects prior to and following blocking: (**a**) effect of matching prior to blocking (the map is on the left, the camera image is on the right, and the numbers in the figure represent incorrect matches); (**b**) effect of matching following blocking (the map is on the left and the camera image is on the right).

In the blocking experiment, Figure 12 presents the matching results of a frame of a UAV aerial image with the map block and unblocked map. It can be observed that the map following blocking presents more matching points with the UAV aerial image, and there is evidence of some incorrect matches (we marked them with black numbers) when not blocking. Table 2 shows the frame rate of the video frame registration in the two ways. It can be observed that the feature-matching process has a higher frame rate after the map is blocked, which improves the speed of registration. Table 3 shows the number of matching-point pairs for 10 randomly selected frames. We presented the larger value in bold and can observe that there were increased numbers of matching points following blocking.

**Figure 13:**
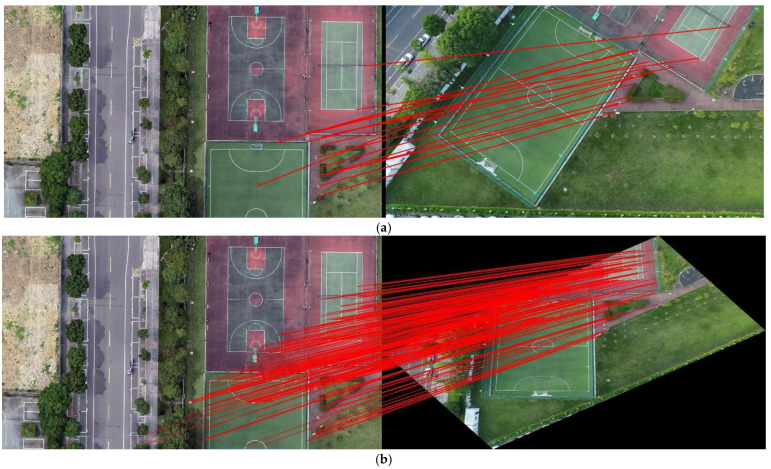
Matching effects prior to and following image rotation: (**a**) matching effect prior to rotation; (**b**) matching effect following rotation.

In the rotation experiment, Figure 13 presents the matching effect of the map with the UAV aerial images prior to and following rotation. In order to better display the results, we removed the matches with a matching-confidence result lower than 0.3, and observed that there were more matching points following the image rotation, and the performance of feature matching was greatly improved. Table 4 depicts the comparison results of the number of matching points in 10 frames of images. The higher values are presented in bold, and we can observe that when the UAV aerial image and map roughly face the same direction, increased matching-point pairs can be obtained.

### 4.3. Comparison Conducted Prior to and Following the Addition of Inter-Frame-Matching Information

This section of the experiment was divided into two parts: one verifies that frame-to-frame matching works better than map-to-frame matching; the second verifies that the stability of video frame registration is greatly improved after integrating inter-frame matching.

**Figure 14:**
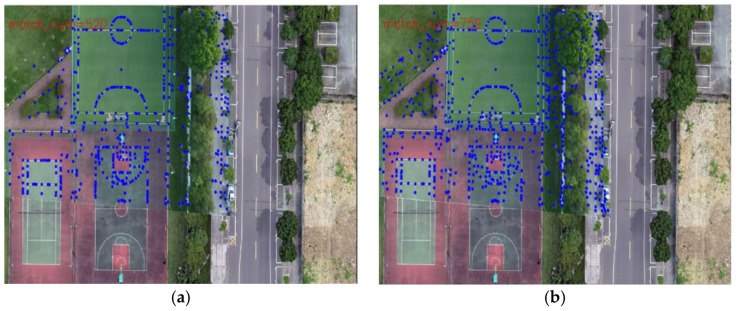
Registration effect: (**a**) match with map; (**b**) match with the transformed previous frame.

**Figure 15:**
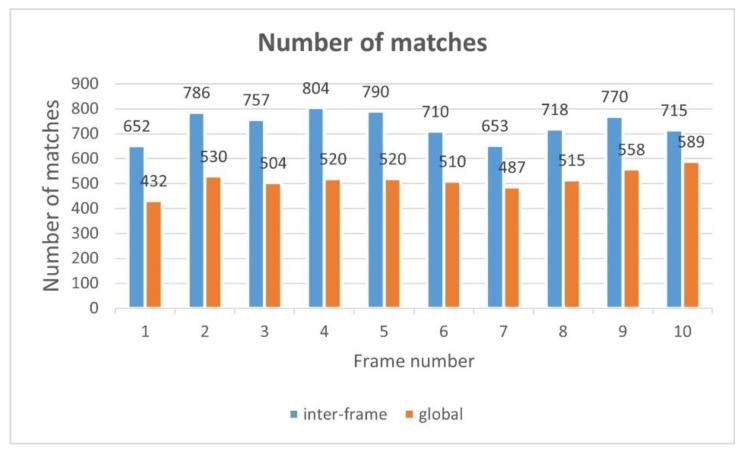
Comparison of the number of matching points with the transformed previous frame and map.

As shown in Figure 14, the blue dots in the image represent matching points. One can observe the richer matching points in the right-hand-side image. In Figure 15, 10 frames are extracted. By comparing the pairs of matching points obtained through the two methods, one can observe that when the UAV aerial image is matched with the transformed previous frame, there are more matching points than when it is matched with the map.

This experiment was conducted to verify that the stability of the registration can be improved by integrating inter-frame-matching behavior. Since the motion between two frames is very reduced, the homography transformation matrices of two adjacent frames should be close to each other during the registration process. The stability can be determined by the difference between the transformation matrices of the two adjacent frames, and the greater the average value of the difference between the transformation matrices of the two adjacent frames, the more unstable the registration process. The difference of homography transformation matrices between two adjacent frames can be obtained by using Equation (11):

(11) $$H_{error}=\sum_{i=1}^{m}\sum_{j=1}^{n}{\left(a_{ij}-b_{ij}\right)}^{2}\;$$

where $a_{ij}$ is the value at position (i, j) of the transformation matrix in the previous frame, $b_{ij}$ is the value at position (i, j) of the transformation matrix in the current frame, m and n represent the row and column of the transformation matrix, respectively, and $H_{error}$ represents the difference between the two matrices.

We recorded a video taken by the tethered UAV, and we registered each frame with the map using two methods: one matched with the map only, and the other integrating inter-frame-matching behavior. A total of 100 frames from the video were selected to save the experimental results, and the $H_{error}$ values of the 100 frames under the two methods were compared.

**Figure 16:**
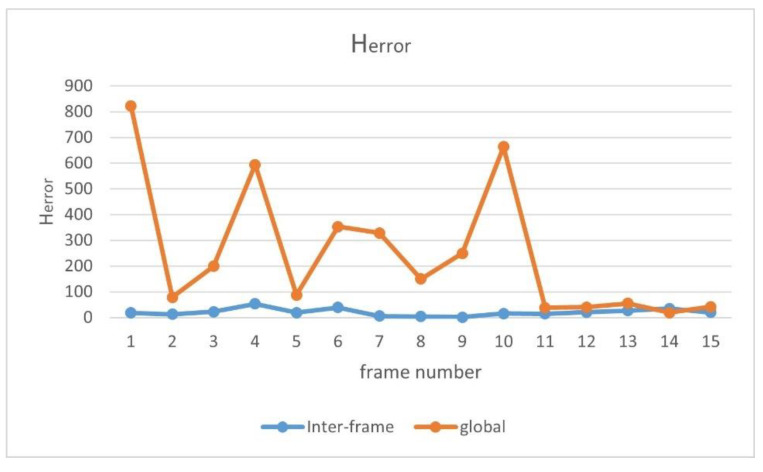
Comparison of the inter-frame and global $H_{error}$ values.

Due to the limited space of the paper, Table 5 only shows the $H_{error}$ values of 15 sampling frames and the average value of 100 frames. The lower values are presented in bold, and it can be observed that the difference between the transformation matrices of the two adjacent frames is very minor after integrating the inter-frame-matching technique, while the difference between the transformation matrices of the two adjacent frames is relatively considerable when the inter-frame-matching technique is not utilized. This shows that incorporating inter-frame-matching techniques into video frame image registration can produce a stable registration result. In Figure 16, we visually present the results we obtained. In the figure, the yellow line represents the result without utilizing inter-frame matching, while the blue line represents the result with the usage of inter-frame matching. It can be observed that after the integration of the inter-frame-matching technique, the transformation matrix between the two adjacent frames presents a minor difference, and the entire video registration process is more stable.

### 4.4. Comparison of the Registration Effects Prior to and Following the Real-Time Update of Map Features

This experiment was designed to verify that a greater registration accuracy can be achieved after updating map features. The experiments were conducted with and without updating the map features. Two scenes were selected for the experiment and the experimental data were collected using the tethered UAV (the video was collected with the camera tilted in order to increase the difficulty of registration). Two methods were used to register each frame of the video in real time.

The transformation matrix can be obtained by the feature-matching technique, and the UAV aerial image can be transformed into the coordinate system of the map through the transformation matrix. There is an overlapping area between the transformed UAV aerial image and the map, and the coincidence degree of the two images can be determined by the difference image of the overlapping area. (The difference image can be obtained by subtracting the gray image of the transformed UAV aerial image from the gray image of the map. That is, the gray values of two corresponding pixels are subtracted.) The pixel value of the difference image represents the difference between the two images at this pixel point. The smaller the average pixel value of the difference image, the greater the accuracy of the UAV aerial image and map registration. In other words, the more white parts there are in the difference image, the worse the accuracy of registration. The average pixel value in the effective area of the difference image can be calculated using the following equation:

(12) $$index=\left(\sum_{i=0}^{rows}\sum_{j=0}^{cols}abs\left(map\left[i,j\right]-frame\left[i,j\right]\right)\right)/flag\;$$

where map represents the grayscale image of the map block, frame represents the grayscale image of the frame image following the homography transformation, *i*, *j* satisfies frame[i,j]!=0, flag is the number of eligible pixels, and index represents the average of the gray value of the effective region in the difference image.

In terms of the result evaluation criteria, we divided the results into subjective and objective evaluations, and for the latter, we used the number of matching points and index value. The experiment was divided into two groups. Due to the limited space of the paper, 13 frames (the 15th, 30th, 45th, 60th, 75th, 90th, 105th, 120th, 135, 150th, 165, 180th, and 195th frames) from the video were selected, and the registration results of these frames under the two methods were evaluated and compared.

#### 4.4.1. Experiment 1 (Group 1)

**Figure 17:**
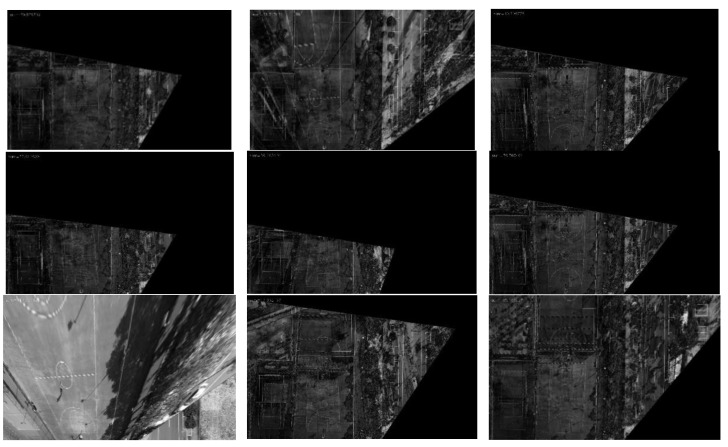
Registration difference image (without updating the map features).

**Figure 18:**
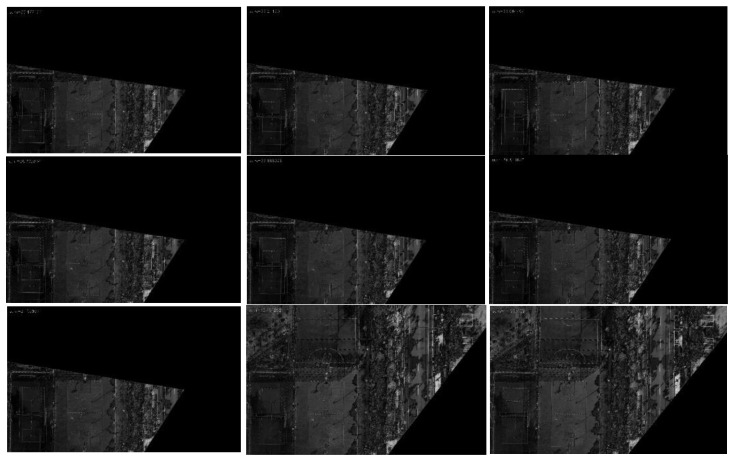
Registration difference image (after updating the map features).

In order to better display the results, we selected 9 frames from the 13 sampling frames to present their graphical results. Figure 17 depicts the difference image of the registration results without updating the map features, and Figure 18 shows the difference image after updating the map features (note: one can observe that the pixel value of the difference image remains high after updating the map features because there are certain differences evident in the color and brightness between the map and UAV aerial images). It can be observed that the top-left-corner areas in the first, third, and eighth images without being updated are whiter than those that have been updated, while the second and seventh images are more obvious, indicating that their registration accuracy is worse.

**Figure 19:**
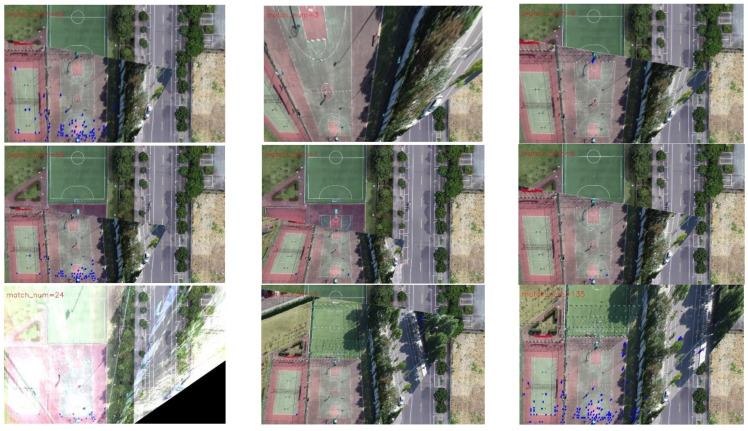
Registration image (without updating the map features).

**Figure 20:**
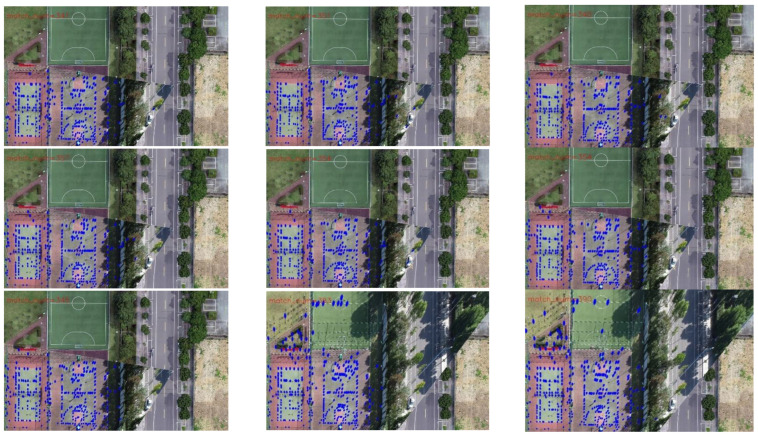
Registration image (after updating the map features).

Figure 19 and Figure 20 present the registration results prior to and following the updating of the map features. It can be observed that the registration results of the second, third, fourth, fifth, seventh, and eighth images present an obvious misalignment without updating the map features. In addition, we can also observe that there are basically no matching points evident when the frame images are matched with the map without updating the features (the blue points in the figure indicate the matching points). However, after updating the map features, the matching points of the image significantly increase.

**Figure 21:**
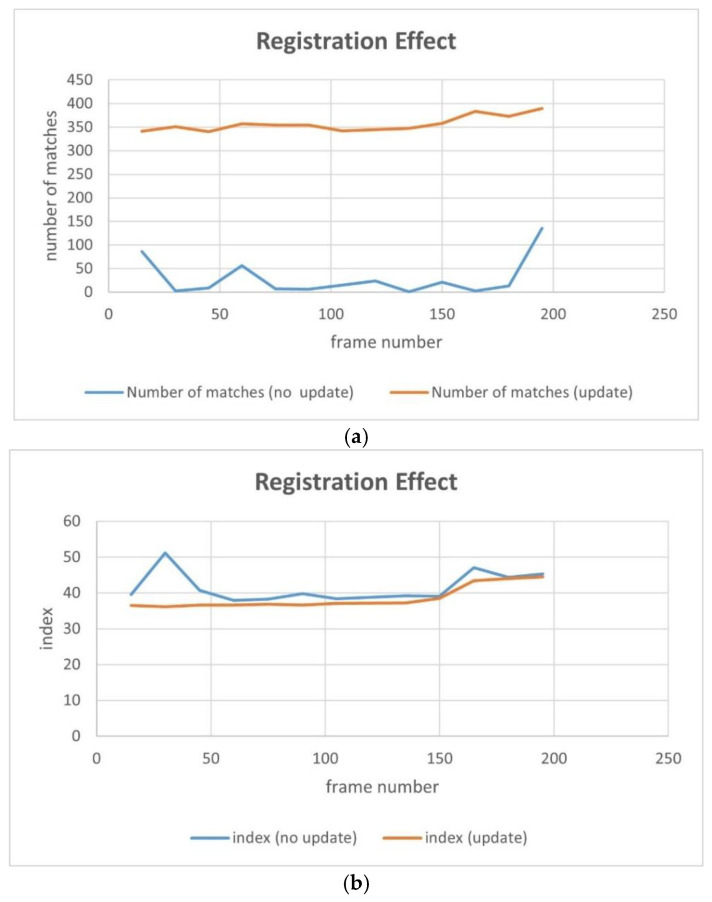
Comparison of the number of matching points and index values: (**a**) number of matches; (**b**) average pixel value of the registered difference image (index ).

Table 6 exhibits the results of the index value and number of matching points of the 13 sampling frames. It can be observed that after updating the map features, the matching points between the UAV aerial image and map significantly increase, and the index value is basically lower than that without updating. Figure 21 presents the results exhibited in Table 6 in a graphical way, and it can be observed that the matching points dramatically increase after updating the map features. Although the change in the index value is not obvious, it attains a smaller value for each frame, which also means that the registration accuracy is higher.

#### 4.4.2. Experiment 2 (Group 2)

**Figure 22:**
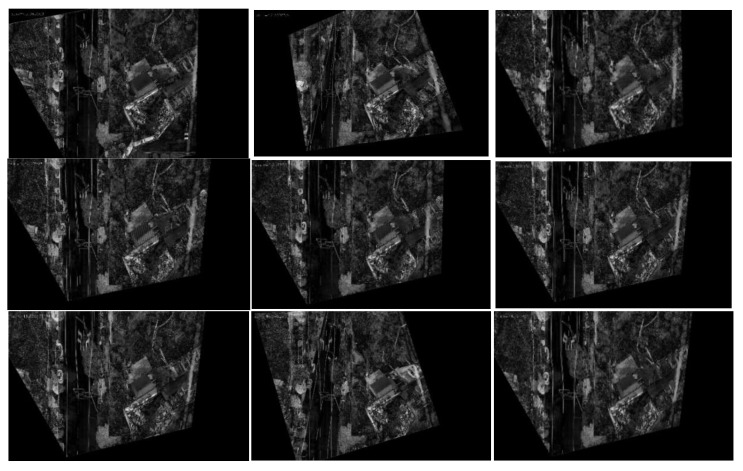
Registration difference image (without updating the map features).

**Figure 23:**
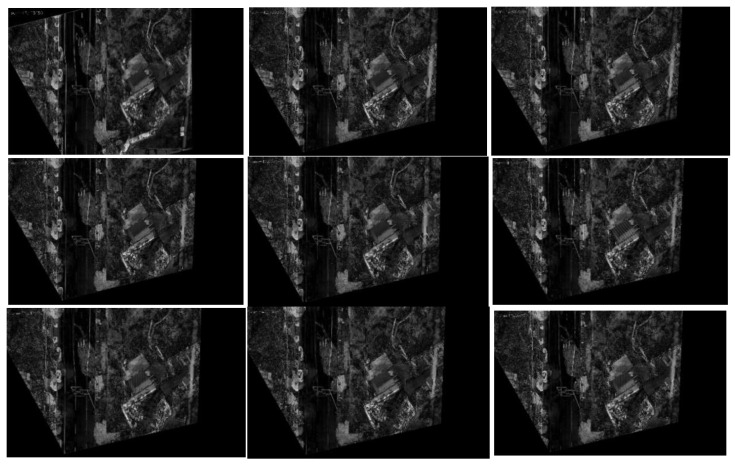
Registration difference image (after updating the map features).

**Figure 24:**
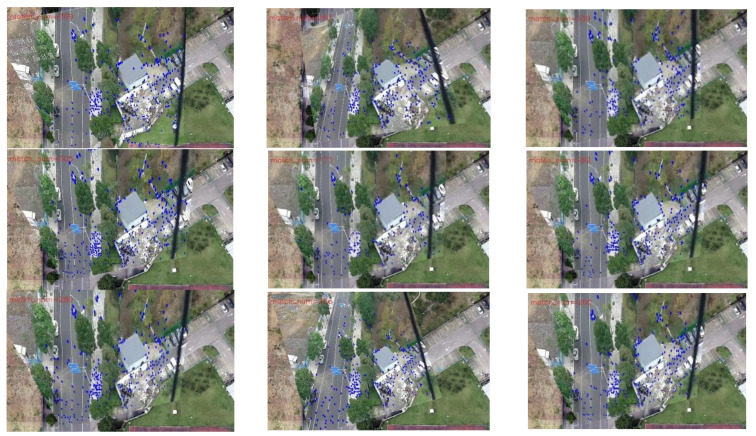
Registration image (without updating the map features).

**Figure 25:**
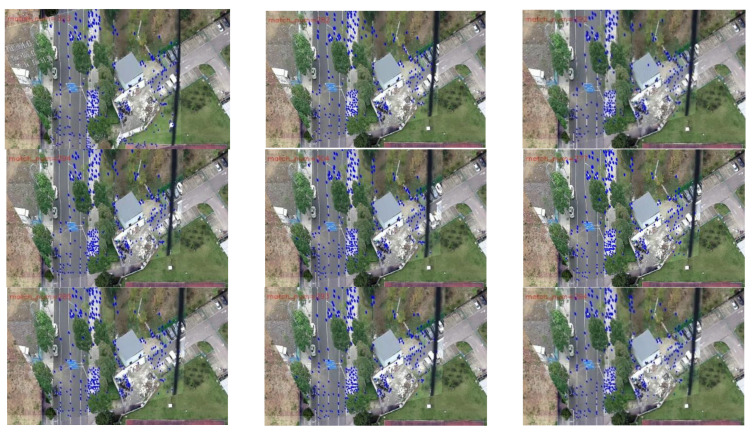
Registration image (after updating the map features).

For the second set of experiments, similarly, we selected 9 from 13 frames for the graphical display; Figure 22 shows the difference image without updating the map feature and Figure 23 shows the difference image after updating the map feature. It can be observed that the lower-left-corner area of the third, fourth, and seventh images without receiving an update are whiter than those that have been updated, and there are obviously incorrect transformations in the second and eighth images. Figure 24 and Figure 25 depict the registration results prior to and following the update of the map feature. It can be observed that when the feature is not updated, the second and eighth registration results present considerable deformations. Although the contrast is not obvious in the first, third, fourth, fifth, sixth, seventh, and ninth images, it can also be observed that the edge of the overlapping area is misaligned.

Table 7 presents the results of the index value and number of matching points of the 13 sampling frames. It can be observed that after updating the map feature, the number of matching points basically increases; however, the increase is less than that of experiment 1, which is caused by the richer texture features of this scene. On the other hand, the index value is basically smaller than that without updating, and the result also is more stable.

**Figure 26:**
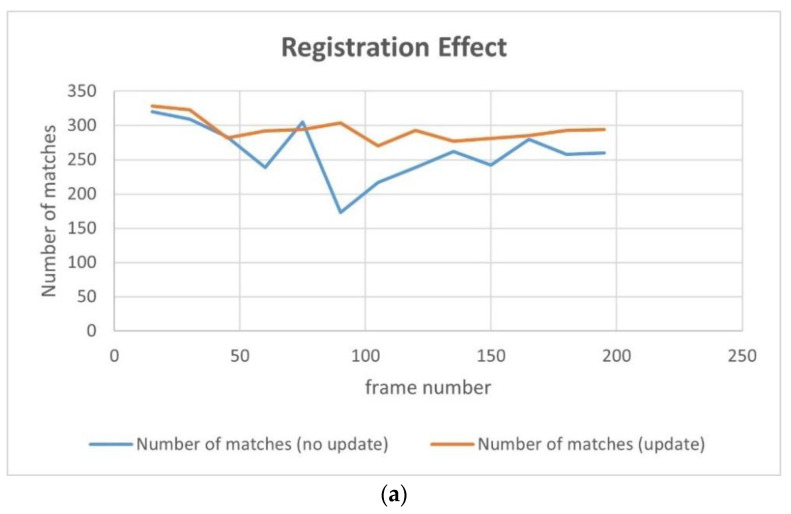

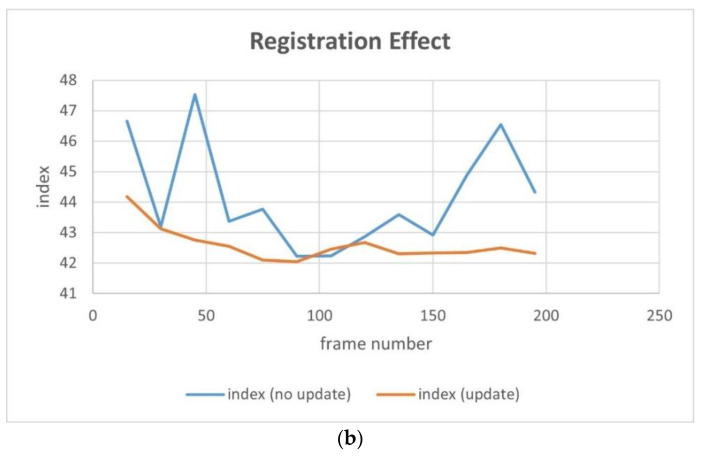
Comparison of the number of matching points and index values: (**a**) number of matches; (**b**) average pixel value of the registered difference image (index ).

Figure 26 shows the results of Table 7 graphically; the yellow line represents the results after updating the map feature and the blue line represents the results without updating the map feature. It can be observed that there are a good number of matching-point pairs prior to and following updating; however, the number of matching points is further improved and tends to be stable, and the index is basically 2–3 points smaller after updating, and the results are relatively stable.

## 5. Discussion

With the rapid development of computer vision and UAV technologies, UAVs are often used in the field for certain tasks, such as visual detection and tracking to analyze or monitor targets; however, this only displays the information of an image and only conveys the visual feeling. If the correspondence between the real-time frames of the UAV and geographic map can be determined, the camera image can be endowed with geographic information. Increased applications can be obtained by transmitting the target geographic information to other platforms, such as combining this with the model map or 3D platform to achieve a virtual reality effect.

In the more ancient work, the projection transformation method was used to project the real-time frame onto the map, and the position of the camera image was calculated by the position information of the UAV and angle information of the camera. However, this method requires the information provided by the UAV to be extremely accurate, and the rotations of the UAV and camera make the calculation process very complex, including numerous accumulated errors and a lack of flexibility. With the gradual development of feature-matching algorithms in the field, both their accuracy and speed have improved; therefore, the improvement of the feature-matching techniques makes it possible to accurately register the UAV aerial images with the map. The UAV aerial images and geographic map are registered by feature matching, so that the UAV aerial images also have geographic coordinates, and the real-time geolocation of the target is realized.

The traditional feature-matching algorithms include Sift [9], Surf [10], Brisk [37], etc. However, they are not real-time methods and can only process a single image; therefore, their application scope is narrow. Therefore, a lot of research has been conducted on speeding up these algorithms, such as meshing or eliminating invalid regions; however, they remain very dissimilar to the real-time method. The emergence of the Orb algorithm has solved the problem of the real-time method, and the Orb algorithm is widely used in various studies because of its superior performance. However, although the Orb algorithm has a good performance, it is difficult to achieve correct matching for scenes with sparse textures, and it even generated a high error rate. In this study, the SuperPoint and SuperGlue algorithms, which exhibit real-time performances, were adopted. The SuperGlue algorithm has a better and more stable performance in relation to sparse texture scenes, and it is suitable for performing feature matching for maps with sparse textures (Figure 10 and Figure 11).

In addition, the map has a wide range, while the UAV aerial image has a narrow range. There is a wide gap in the scale between the two methods; therefore, it is difficult to perform feature matching between them. The easiest way to solve this problem is to cut the map; however, the UAV aerial image is constantly changing. Thus, how do we attain the appropriate map following the blocking process? The traditional method used in the field is to obtain the position directly below the UAV through the GPS information of the UAV to select the corresponding map block. On this basis, we used the pan–tilt–zoom camera and introduced the rotation information of the camera, so that our method could register the UAV aerial images under the tilt angle. In addition, our method could flexibly rotate the camera image by the heading angle of the UAV, so that the UAV aerial images with different angles could also be registered in the study (Figure 12 and Figure 13).

The movement of the UAV and rotation of the camera caused the scene to be changeable; however, the map was immutable, which may cause the performance of the feature-matching algorithm to be unstable and may achieve poor results for some complex scenes. Inspired by the idea of real-time mapping, we proposed a method to update the map feature in real time, so that the map could change according to the change in the external environment. The experiments (Figure 17, Figure 18, Figure 19, Figure 20, Figure 21, Figure 22, Figure 23, Figure 24, Figure 25 and Figure 26) showed that, in some scenes where the feature-matching performance was difficult, the proposed method effectively improved the accuracy of the feature matching and presented greater robustness and flexibility. In addition, the proposed method combined global and inter-frame matching techniques to create a more stable registration process, the inter-frame matching technique reduced the fluctuation of the global-matching technique, and the global matching technique restricted the cumulative error produced by the inter-frame matching method, as shown in Figure 16.

Indeed, the proposed method also had some limitations. When the camera tilt angle was very large, it produced poor results, and the frames and prior UAV data were required to be collected synchronously and have a low-delay frequency. In future research, we hope to optimize the existing problems in this regard.

## 6. Conclusions

Due to the sparse texture and wide coverage of the map, as well as the large difference between the dynamic UAV aerial image and the static map, it is difficult to accurately register the UAV aerial image and the map using the traditional feature-matching algorithm. To solve this problem, in this study, the SuperPoint and SuperGlue algorithms, which are based on deep learning, were used for feature matching. The hierarchical blocking strategy, combined with prior UAV data, was introduced to improve the matching performance, and matching information obtained between frames was introduced to render the registration process smoother and more stable. The concept of updating the map features with UAV aerial image features was proposed with the aim of updating the map features in real time, rendering the method more adaptable to the changing environment and improving the registration accuracy and the robustness and applicability of the algorithm. Finally, the UAV aerial image can be accurately registered on the map in real time, adapting to the changes in the environment and the camera head. A large number of experiments showed that the proposed algorithm is feasible, practical, and scientific and has specific application value in the fields of UAV aerial image registration and UAV aerial image target geo-positioning.

## Figures and Tables

**Table 1:** Comparison of the algorithms’ matching accuracy.

Experimental Group	Algorithm	Number of Matches	Accurate Number	Accuracy Rate
1	Orb	190	10	0.05
	SuperGlue	583	274	0.47
2	Orb	397	16	0.04
	SuperGlue	1706	1283	0.75

**Table 2:** Real-time performance of the algorithms.

Strategy	Algorithm	Frame Rate
Non-blocking	SuperGlue	9
Blocking	SuperGlue	12

**Table 3:** Number of the matching points before and after blocking. The better results are highlighted in bold.

Sampling Frame	1	2	3	4	5	6	7	8	9	10
Non-blocking	7	55	14	26	41	25	4	5	96	64
Blocking	**282**	**314**	**88**	**166**	**95**	**105**	**136**	**87**	**400**	**242**

**Table 4:** Number of the matching points before and after rotation. The better results are highlighted in bold.

Sampling Frame	1	2	3	4	5	6	7	8	9	10
Before Rotation	56	70	71	35	91	113	22	30	60	41
After Rotation	**274**	**332**	**334**	**178**	**452**	**496**	**147**	**198**	**225**	**208**

**Table 5:** Comparison of the inter-frame and global $H_{error}$ values. The better results are highlighted in bold.

Sampling Frame	1	2	3	4	5	6	7	8	9	10	11	12	13	14	15	Aver_ Value
Inter-frame	**18**	**12**	**22**	**53**	**19**	**39**	**5**	**4**	**1**	**15**	**14**	**20**	**27**	34	**20**	22.7
Global	821	79	199	594	87	352	328	150	249	663	38	39	55	**19**	42	226

**Table 6:** Comparison of the number of matching points and index values. The better results are highlighted in bold.

Frame Number	Update	15	30	45	60	75	90	105	120	135	150	165	180	195
Number of matches	Yes	**341**	**351**	**340**	**357**	**354**	**354**	**342**	**345**	**347**	**358**	**383**	**373**	**390**
	No	86	3	9	56	7	6	15	24	1	21	3	13	135
Index	Yes	**36.5**	**36.2**	**36.6**	**36.7**	**36.9**	**36.6**	**37.1**	**37.1**	**37.3**	**38.5**	**43.5**	**44.0**	**44.5**
	No	39.6	51.2	40.7	37.9	38.3	39.8	38.4	111.7	39.2	39.0	47.0	44.4	45.3

**Table 7:** Comparison of the number of matching points and index values. The better results are highlighted in bold.

Frame Number	Update	15	30	45	60	75	90	105	120	135	150	165	180	195
Number of matches	Yes	**328**	**323**	282	**292**	294	**304**	**270**	**293**	**277**	**281**	**285**	**293**	**294**
	No	320	309	**283**	239	**305**	173	217	239	262	242	280	258	260
Index	Yes	**44.1**	**43.1**	**42.7**	**42.5**	**42.1**	**42.0**	42.4	**42.6**	**42.3**	**42.3**	**42.3**	**42.5**	**42.3**
	No	46.6	43.1	47.5	43.3	43.7	42.2	**42.2**	42.8	43.5	42.9	44.8	46.5	44.3

## Data Availability

The data presented in this study are available on request from the author.
